# Pool dynamics of time-dependent compartmental systems with application to the terrestrial carbon cycle

**DOI:** 10.1098/rsif.2022.0843

**Published:** 2023-03-22

**Authors:** George Chappelle, Alan Hastings, Martin Rasmussen

**Affiliations:** ^1^ Department of Mathematics, Imperial College London, 180 Queen’s Gate, London SW7 2AZ, UK; ^2^ Department of Environmental Science and Policy, University of California, Davis, CA 95616, USA; ^3^ Santa Fe Institute, 1399 Hyde Park Road, Santa Fe, NM 87501, USA

**Keywords:** carbon cycle, CASA model, compartmental system, McKendrick–von Foerster equation, non-autonomous dynamical system, transit time

## Abstract

Compartmental models play an important role to describe the dynamics of systems that involve mass movements between different types of pools. We develop a theory to analyse the average ages of mass in different pools in a linear compartmental system with time-dependent (i.e. non-autonomous) transfer rates, which involves transit times that characterize the average time a particle has spent in a particular pool. We apply our theoretical results to investigate a nine-dimensional compartmental system with time-dependent fluxes between pools modelling the carbon cycle which is a modification of the Carnegie–Ames–Stanford approach model. Knowledge of transit time and mean age allows calculation of carbon storage in a pool as a function of time. The general result that has important implications for understanding and managing carbon storage is that the change in storage in different pools does not change monotonically through time: as rates change monotonically a pool which initially shows a decrease may then show an increase in storage or vice versa. Thus caution is needed in extrapolating even the direction of future changes in storage in carbon storage in different pools with global change.

## 1. Introduction

Compartmental systems are important models for many biological systems ranging from pharmacokinetics to ecology [1–3]. We are mainly motivated by the terrestrial carbon cycle, where a description of the dynamics of carbon is typically presented in terms of the amount of carbon in different pools and the fluxes between the pools. Often density-dependent and time-dependent aspects are ignored and, in the simplest description, the carbon cycle is modelled by a linear and autonomous compartmental system.

It is increasingly becoming clear that the fluxes of carbon between pools are changing relatively rapidly in the face of various aspects of global change. These changes are mainly driven by increases in temperature, which lead to increasing rates of soil decomposition [4–6]. Consequently, in corresponding models, constant parameters need to be replaced by time-dependent functions [7–9], leading to non-autonomous compartmental systems (meaning that parameters change through time in contrast to autonomous systems with parameters that are constant in time). Of great interest is understanding the dynamics of carbon over time in response to these changes; the analysis of these models thus needs to make use of tools from the theory of non-autonomous dynamical systems [10]. We note that many other systems require a similar mathematical description incorporating time-dependence. As an example, the movement of dissolved material between different aquatic pools depends on time, since the fluxes change in the face of changes in hydrology.

Regarding the carbon cycle, one challenge is how to increase overall carbon storage in various terrestrial pools. A fundamental step to address this challenge concerns the understanding of the transit times of carbon involved, which is the mean time a carbon particle spends in the compartmental system (or some part of it) measured as the mean time from entry into the system to leaving the system. In a well-mixed system, increasing transit time will increase the storage in that pool, while decreasing transit time will decrease storage. It is thus of utmost interest to understand how the transit time in different pools changes as fluxes between pools are changed. The appropriate timescales would be ones with response times in decades. Often, short-term trends are observed and a significant question is how reasonable it is to project trends into the future when conditions are changing. Under what conditions will short-term trends continue? Can we predict general features that lead to changes over longer time periods from knowledge of how fluxes are changing through time?

The question of transit times is classical and well studied in the framework of autonomous compartmental systems (see [11–14], and see [15] for an overview about the literature and applications to the terrestrial carbon cycle). Transit times for non-autonomous compartmental systems have only been recently studied: in our earlier papers [16,17], we develop a theory for non-autonomous transit times and mean ages, which was extended in [18] to particular nonlinear systems and to higher-order moments of transit time distributions (see also [14], chapter 3). In this paper, we build on our previous works, and we provide a more detailed transit time analysis which gives additional information regarding the average times spent in different pools rather than (only) in the whole system. We would like to stress that, although our primary motivation comes from the terrestrial carbon cycle, the main results in this article are of a theoretical nature and applicable to many different systems. We note that non-autonomous transit times have been applied to understand the dynamics of vesicles [19], and there have been recent advances regarding non-autonomous transit times in applications to hydrology (which are not modelled as compartmental systems), see the recent review article [20] and references therein.

Generalizing the classical autonomous theory of transit times to non-autonomous compartmental systems is not straightforward. We stress that due to the time-dependent nature, in contrast to the autonomous case, it makes a difference whether we consider the past or the future. More precisely, when considering the past, a meaningful notion of transit time describes the mean age of particles that leave the system at a particular time (hence, only information from the past is used). By contrast, when considering the future, a meaningful notion of transit time describes the average time a particle stays in the system from the time it entered the system. Note that it depends on the application which approach is more suitable. The transit times considered in this paper relate to the past of the system, and it is also important to realize that this does not prevent predictions for the future. In fact, in this paper, we consider an application to a nine-dimensional model of the terrestrial carbon cycle, which considers increases of atmospheric CO_2_ in the future, and we consider transit times up to the year 2450, which incorporates information until the year 2450 (but not beyond). We note that in our paper [17], we develop a theory of pool transit times that depend only on the future, and we apply this to understand typical times spent in certain breeding stages of the Southern fulmar. In such a context, the past approach is not possible, because we focus on one individual, in contrast to the many carbon atoms that leave the system at a specific time.

This paper is organized as follows. We begin in the next section illustrating the ideas with the simplest compartmental system, one with two compartments. We start with the constant in time case and then move on to the case where underlying conditions can change, a non-autonomous system. After illustrating the complications that can arise even in simple systems, we move on to a general mathematical theory for linear non-autonomous compartmental systems. In §3, we introduce the pool mean age equation, which is an ordinary differential equation that tracks the mean times particles spend in certain parts of the compartmental systems. We use information obtained from the pool mean age equation to define the pool transit times in §4. We then apply our theoretical results in §5 to a system modelling terrestrial carbon under climate change.

## 2. Motivation

### 2.1. The autonomous case

Movements of mass between different pools can be modelled by a linear autonomous compartmental system, which is a linear differential equation of the form

$$\dot{x}=Bx+s,$$

where *B* = (*b*_*ij*_) is a *d* × *d* matrix and *s* is a *d*-dimensional vector. Due to mass conservation, the matrix *B* (the so-called *compartmental matrix*) and the vector *s* have certain properties. Note that the *i*th row of the matrix *B* represents the behaviour in the *i*th pool, and *b*_*ij*_ is the rate at which mass moves from pool *j* to pool *i*, whereas *b*_*ii*_ is the rate (mass per time) at which mass leaves the pool *i* (this includes transfer to other pools and losses from the system). For this reason, *b*_*ii*_ needs to be negative, and *b*_*ij*_ needs to be non-negative for i≠j. In addition, the column sums $\sum_{j}b_{ij}$ are zero if no mass leaves the system directly from pool *i* and negative if mass leaves the system from pool *i*. We also require the matrix *B* to be invertible. This implies that the column sum $\sum_{j}b_{ij}$ is negative for at least one column *i*. The vector *s* has non-negative entries, since it describes the rate of mass that enters the compartmental system from outside.

Consider a compartmental system with the coefficient matrix

$$B=\left(\begin{array}{cc} -1 & 0\\ \frac{1}{2} & -\frac{1}{4} \end{array}\right).$$

Here mass can move from pool 1 to pool 2 (*b*_21_ > 0), but not from pool 2 to pool 1 (*b*_12_ = 0). Mass leaves pool 1 with the exponential rate *b*_11_ = −1, and half of the mass that leaves goes into pool 2 (since *b*_21_ = −(1/2)*b*_11_), while the other half leaves the system. Mass in pool 2 leaves with the exponential rate *b*_22_ = −0.25, and all mass leaving this pool leaves the system (since *b*_12_ = 0).

We aim at determining characteristic times that describe how long mass stays in which pool. It is well known that these times can be read from the negative of the matrix inverse −*B*^−1^ = (*c*_*ij*_) [1]. One possible interpretation of these characteristic times is that, when the system is in equilibrium (i.e. when *x* = −*B*^−1^*s*), for a particle that leaves the system, the entries of the negative matrix inverse describe the average times this particle has spent in the different pools. More precisely, if mass comes from the outside only into pool *j*, then the average time spent in pool *i* of a leaving particle is given by *c*_*ij*_.

The negative inverse of the above matrix is given by

$$-B^{-1}=\left(\begin{array}{cc} 1 & 0\\ 2 & 4 \end{array}\right),$$

and thus, when particles only come into pool 1 (e.g. when $s={\left(1,0\right)}^{⊤}$), an average particle that leaves the system has spent one unit of time in pool 1 and two units of time in pool 2. On the other hand, when particles come only into pool 2 (e.g. when $s={\left(0,1\right)}^{⊤}$), then a leaving particle has spent on average four units of time in pool 2 and no time in pool 1.

### 2.2. The non-autonomous case

In this paper, we aim at extending this theory to non-autonomous compartmental models. Non-autonomous compartmental systems are given by differential equations of the form

$$\dot{x}=B(t)x+s(t),$$

where the matrix *B* and the input vector *s* now depend explicitly on time. In the same way as in the autonomous case, the sign of the elements of a compartmental system satisfies certain properties.

We note that corresponding analogues to the elements of the above matrix inverse can only be obtained in a completely different way, and the time-dependent matrix inverses *B*(*t*)^−1^ have no meaning in this different setting.

The main tool to generalize the theory to the non-autonomous case is given by a time-dependent matrix *A*(*t*) = (*a*_*ij*_(*t*)), whose elements *a*_*ij*_(*t*) describe the average time a particle that is currently (i.e. at time *t*) in pool *i* has spent in pool *j* (since entering the system). Here the average is taken over all particles. We note that the quantities *a*_*ij*_, the so-called *pool occupancy times*, are determined by the past of the system, and we will see in a moment that *a*_*ij*_(*t*) = *a*_*ij*_ and *c*_*ij*_ are not equal in general when applied to an autonomous compartmental system. In order to provide appropriate non-autonomous generalizations of *c*_*ij*_, we look at so-called pool-dependent transit times *R*_*i*_(*t*), which are computed using the times *a*_*ij*_(*t*) and describe the average time spent in pool *i* (since entering the system) of a particle that leaves the system at time *t*.

We analyse this in the context of the above (autonomous) example. It should be noted that the transit times *R*_*i*_(*t*) depend on *s*(*t*), in contrast to the quantities *c*_*ij*_ in the autonomous case. We first consider *s*(*t*) = (1, 0), and our theory explained later in this article shows that the matrix *A*(*t*) = *A* is given by

2.1 $$A=\left(\begin{array}{cc} 1 & 0\\ 1 & 4 \end{array}\right).$$

This means that a particle currently in pool 1 has spent on average one unit of time in pool 1 and no time in pool 2. The second row of this matrix explains that a particle currently in pool 2 has spent on average one unit of time in pool 1 and four units of time in pool 2. To compute the transit time *R*_*i*_(*t*) for each pool, one needs to know the amount of mass in each pool, since this determines how much mass will be released from each pool at time *t*. We assume that the system is in equilibrium, and one can compute (*x* = −*B*^−1^*s*) that the mass in pool 1 is given by *x*_1_ = 1, and the mass in pool 2 is given by *x*_2_ = 2. Since mass from pool 1 leaves the system with rate −1/2 and mass from pool 2 leaves the system with rate −1/4, this implies that at a particular time, 50% of mass that leaves comes from each pool. Using the above matrix *A*, this implies that the average time a particle that leaves has spent in pool 1 is given by *R*_1_(*t*) = 1 (here 1 = (1/2) · 1 + (1/2) · 1, corresponding to first column of the matrix *A*), while the average time a particle that leaves has spent in pool 2 is given by *R*_2_(*t*) = 2 (here 2 = (1/2) · 0 + (1/2) · 4, corresponding to the second row of the matrix *A*), and these two numbers correspond to the first column of the matrix −*B*^−1^.

Similarly, in case of *s*(*t*) = (0, 1), one gets

$$A=\left(\begin{array}{cc} 0 & 0\\ 0 & 4 \end{array}\right),$$

and it follows that *R*_1_(*t*) = 0 and *R*_2_(*t*) = 4, which corresponds to the second row of the matrix −*B*^−1^.

We now apply this idea to the specific non-autonomous compartmental system

2.2 $$\dot{x}=B(t)x+s,$$

where

$$B(t)=\left(\begin{array}{cc} -1 & 0\\ \frac{1}{2} & -\frac{1}{5}+\frac{1}{10\pi }\arctan(t) \end{array}\right)\;\text{and}\;s=\left(\begin{array}{c} 1\\ 0 \end{array}\right).$$

This system no longer depends on time in the limits *t* → −∞ and *t* → ∞, and we get the limiting matrices *B*_−∞_ = lim _*t*→−∞_ *B*(*t*) = *B*, where *B* is the matrix from above, and

$$B_{\infty }=\left(\begin{array}{cc} -1 & 0\\ \frac{1}{2} & -\frac{3}{20} \end{array}\right)\;\text{with}\;-B_{\infty }^{-1}=\left(\begin{array}{cc} 1 & 0\\ \frac{10}{3} & \frac{20}{3} \end{array}\right).$$

The behaviour of the above non-autonomous system will be close to the compartmental system $\dot{x}=B_{-\infty }x+s$ for times *t* ≪ 0 and close to the system $\dot{x}=B_{\infty }x+s$ for times *t* ≫ 0. In particular, for times *t* ≪ 0, mass leaving the system has spent (on average) *R*_1_(*t*) ≈ 1 unit of time in pool 1 and *R*_2_(*t*) ≈ 2 units of time in pool 2, while for times *t* ≫ 0, mass leaving the system has spent *R*_1_(*t*) ≈ 1 unit of time in pool 1 and *R*_2_(*t*) ≈ 10/3 units of time in pool 2 (as the first column of the matrix $-B_{\infty }^{-1}$ shows; note that mass enters only into pool 1).

**Figure 1.**
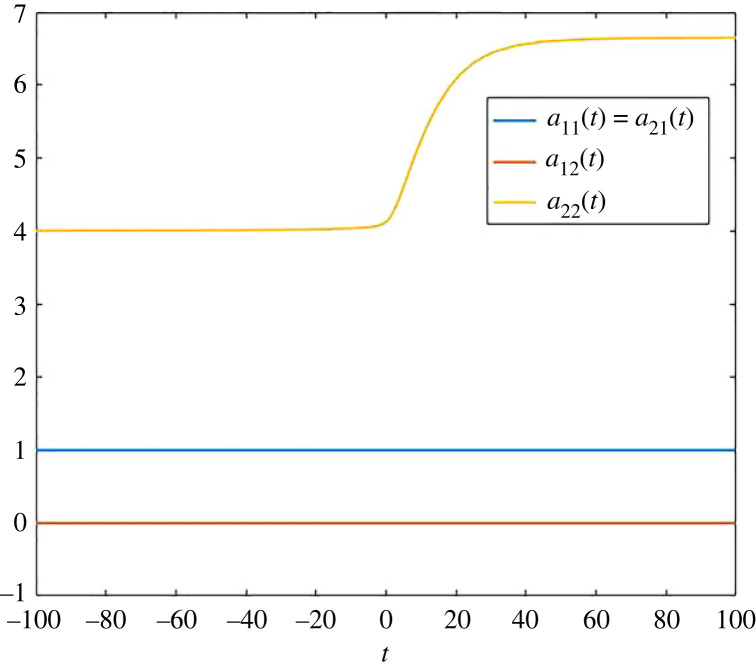
Pool occupancy times *a*_11_(*t*), *a*_12_(*t*), *a*_21_(*t*) and *a*_22_(*t*) for the compartmental system (2.2).

In this paper, we provide non-autonomous analogues for the transit times *R*_1_(*t*) and *R*_2_(*t*) for all times *t*, and not only for those times that are close to −∞ or ∞. These analogues are based on specific pool occupancy times, which we have computed for this example, see figure 1. Recall that the pool occupancy time *a*_*ij*_(*t*) describes the average time a particle that is at time *t* in pool *i* has spent in pool *j*. Here, for *t* ≪ 0, we see that these times correspond to the times described in the matrix *A* in (2.1), which is very reasonable, since these are the pool occupancy times for the limiting system for *t* → −∞. The time-dependent changes in the compartmental system (2.2) only concern the behaviour in pool 2, and escape from pool 2 slows down. As a consequence, we see in the figure that *a*_22_(*t*) increases, and all other pool occupancy times stay constant (also because there is no transfer from pool 2 to pool 1).

**Figure 2.**
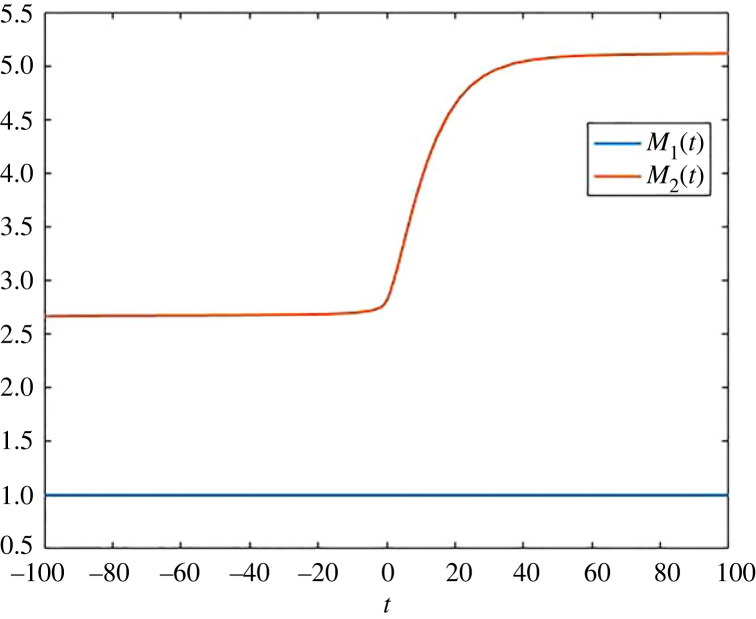
Average times *M*_1_(*t*) and *M*_2_(*t*) as a function of time for the compartmental system (2.2).

In addition to the detailed pool occupancy times *a*_*ij*_, it is often helpful to consider the average time that a particle in the whole system has spent in a specific pool. To obtain these quantities, we get weighted times of the form

$$M_{1}(t)=\frac{a_{11}(t)x_{1}(t)+a_{21}(t)x_{2}(t)}{x_{1}(t)+x_{2}(t)}$$

and

$$M_{2}(t)=\frac{a_{12}(t)x_{1}(t)+a_{22}(t)x_{2}(t)}{x_{1}(t)+x_{2}(t)},$$

and these times are plotted in figure 2.

**Figure 3.**
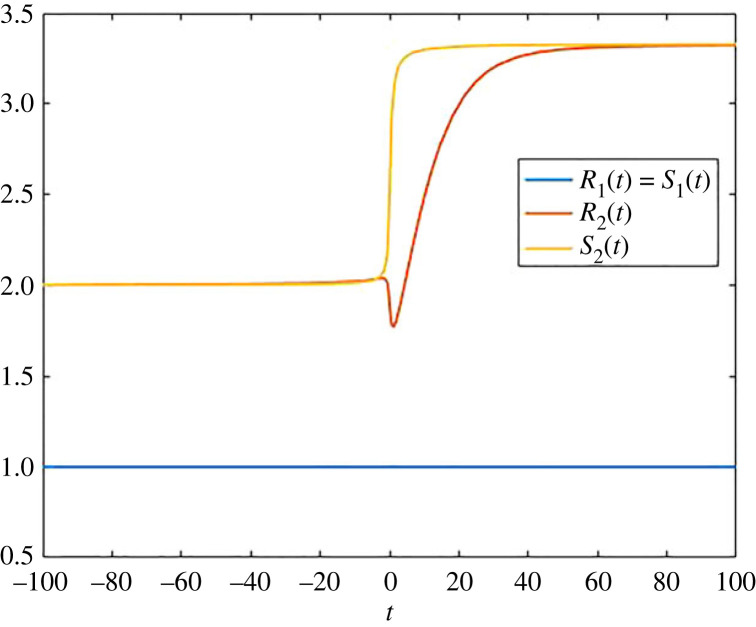
Transit times *R*_1_(*t*) and *R*_2_(*t*) for the compartmental system (2.2), and the corresponding instantaneous autonomous quantities *S*_1_(*t*) and *S*_2_(*t*), which are elements of the first column of −*B*(*t*)^−1^.

We have also computed the transit times for this example, and this is demonstrated in figure 3, in which we also compared this with the corresponding instantaneous autonomous quantities *S*_1_(*t*) and *S*_2_(*t*), coming from the first column of −*B*^−1^(*t*) (note that it is the first column, since *s*(*t*) = (1, 0)). We have plotted in figure 3 the two entries *S*_1_(*t*) (blue line) and *S*_2_(*t*) (yellow line) of the first column of this matrix. The non-autonomous changes slow down the release of particles from pool 2. This in turn means that for a frozen time *t*, the corresponding transit times grow, and this is monotonic, since the slowing is also monotonic. Interestingly, the corresponding behaviour of the non-autonomous system (given by the red line) is non-monotonic. This is due to the fact that at the beginning of the slowing down, fewer particles leave pool 2, in contrast to particles leaving pool 1, and as a consequence, the average time that a particle that leaves at this time has spent in pool 2 decreases. As another consequence of the slowing down, mass increases in pool 2, and then, at some point, the average time a particle that leaves has spent in pool 2 increases, and in the limit *t* → ∞, we reach 10/3.

We have seen that for this example, we could understand the quantities in figure 1 very well, while it was more complicated to explain why we have a non-monotonic behaviour in figure 3 for this very simple example. We now present a more complicated example for which the non-autonomous changes are more difficult to understand. The example is given by the differential equation

2.3 $$\dot{x}=B(t)x+s,$$

where

$$B(t)=\left(\begin{array}{cc} -1+\frac{2}{5\pi }\arctan(t) & 0\\ \frac{1}{2} & -\frac{1}{5}-\frac{1}{5\pi }\arctan(t+10) \end{array}\right)$$

and *s* = (1, 0). By contrast to the above example, both exponential rates in pools 1 and 2 depend on time, and these changes are most pronounced at a different time (note that we have *t* + 10 in the arctan function describing the change in the second pool).

**Figure 4.**
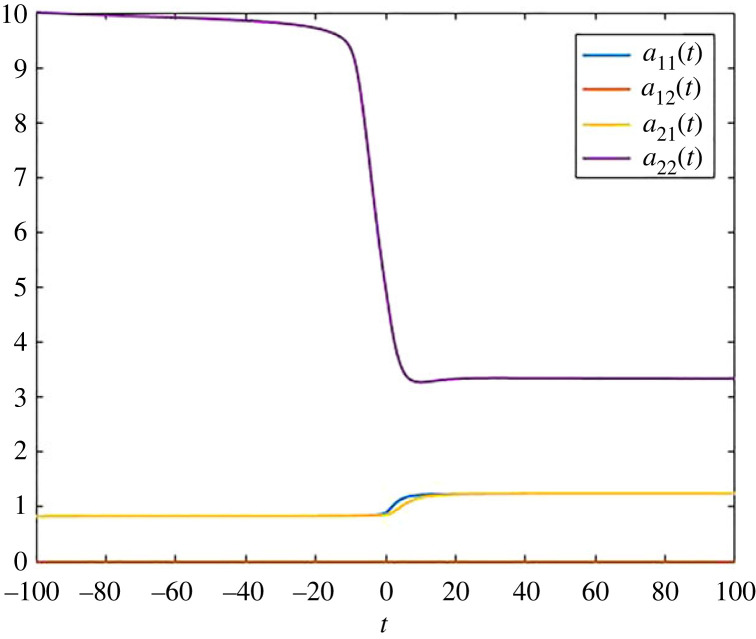
Pool occupancy times *a*_11_(*t*), *a*_12_(*t*), *a*_21_(*t*) and *a*_22_(*t*) for the compartmental system (2.3).

**Figure 5.**
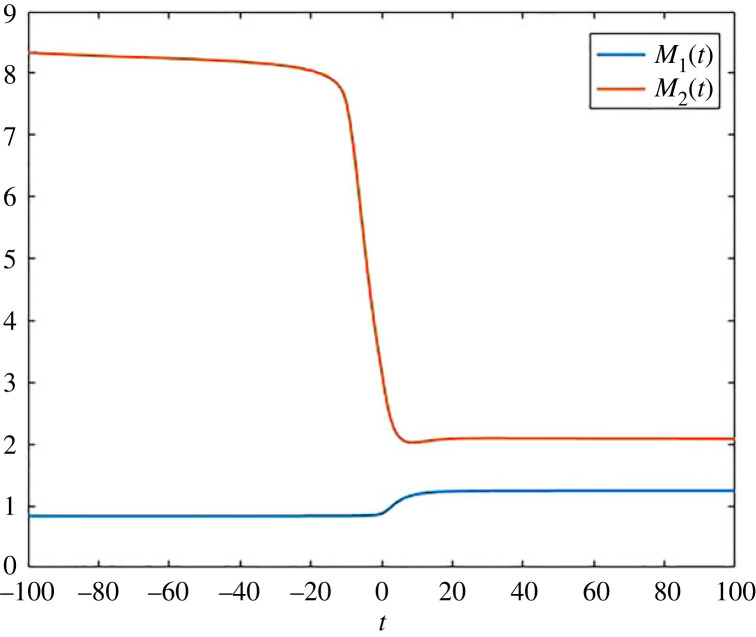
Average times *M*_1_(*t*) and *M*_2_(*t*) for the compartmental system (2.3).

**Figure 6.**
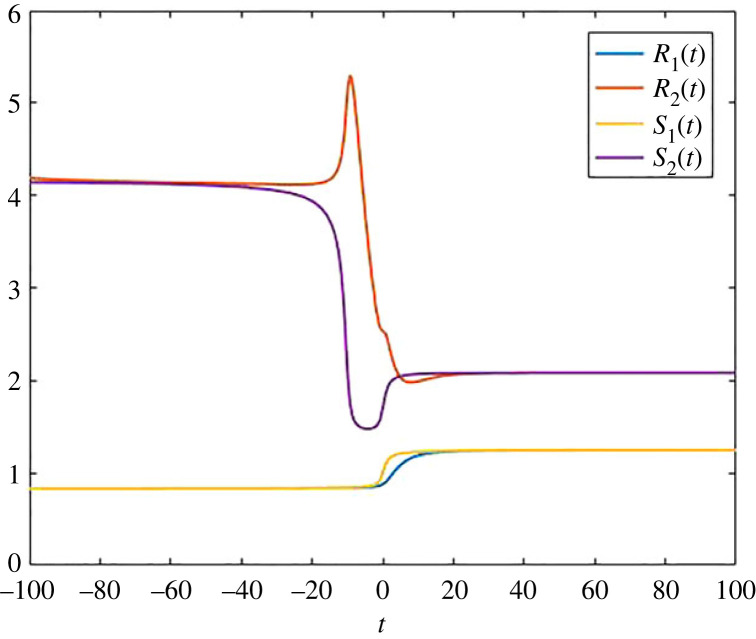
Transit times *R*_1_(*t*) and *R*_2_(*t*) for the compartmental system (2.3), and the corresponding instantaneous autonomous quantities *S*_1_(*t*) and *S*_2_(*t*), which are elements of the first column of −*B*(*t*)^−1^.

The pool occupancy times are given in figures 4 and 5, and we have plotted the transit times in figure 6. We note the pool occupancy times behave like we expect, given by the monotonic changes in the exponential decay rates in both pools. However, even in this relatively simple two-dimensional compartmental system, it is difficult to explain the outcome of the numerical simulation for the transit times presented in figure 6. In particular, we see that the behaviour of *R*_2_ is very non-monotonic and has two different peaks. In general, for more complicated non-autonomous models, the explanation of why transit times change in a certain way is very difficult to understand, and one has to rely on the computations of the quantities we present in this article. We note that pool occupancy times are more predictable, since they fully depend on the past, while transit times both depend on the past (via the pool occupancy times), but also on the output at a specific time, which is given by the model and represented by the matrix *B*(*t*), which allows instantaneous changes.

## 3. The pool mean age equation

In this section, we aim at understanding how the pool occupancy times of the particles develop in the different pools. Here we consider a *d*-dimensional non-autonomous linear compartmental system

3.1 $$\dot{x}=B(t)x+s(t),$$

and we denote by *a*_*ik*_(*t*), as introduced before, the average time spent in pool *k* of a particle that at time *t* is in pool *i*. We demonstrate that the quantities *a*_*ik*_(*t*) are solutions to an ordinary differential equation, the so-called pool mean age equation.

In our earlier paper [16], we have derived a result for accumulations of these quantities. More precisely, with $a_{i}(t)=\sum_{k=1}^{d}a_{ik}(t)$, we get the mean age of a particle that is in pool *i* at time *t* (here age is defined as elapsed time since entering the system from outside via *s*(*t*)), and we showed that the mean ages $a(t)=(a_{1}{\left(t),\ldots ,a_{d}(t)\right)}^{⊤}$ satisfy a non-autonomous differential equation, given by

3.2 $$\dot{a}=g(t,x,a),$$

see ([16], theorem 2). Here the coordinate functions of the right-hand side *g* read as

$$g_{i}(t,x,a)=1+\frac{\sum_{\;j=1}^{d}(a_{j}-a_{i})b_{ij}(t)x_{j}(t)-a_{i}s_{i}(t)}{x_{i}(t)},$$

and note that $x(t)={\left(x_{1}(t),\ldots ,x_{d}(t)\right)}^{⊤}$ is a given fixed solution to the compartmental system (3.1).

This result was derived from the evolution of age distributions, given by the well-known McKendrick–von Foerster equation [21–23], a linear first-order partial differential equation. We now use an extension of the McKendrick–von Foerster equation to derive a differential equation for the more refined average times, given by the pool occupancy times *a*_*ik*_(*t*).

Let *p*_*ik*_(*a*, *t*) be the mass density function on occupancy time *a* spent in pool *k* for the mass in pool *i*, where the occupancy time is the time (spent in this specific pool) since the mass entered the system. Each particle has a (global) age which describes the time since entering the system, and, by looking at the times spent in different pools, this age is the sum of *d* different occupancy times spent in the *d* different pools. We note that $x_{i}(t)=\sum_{k=1}^{d}\int_{0}^{\infty }p_{ik}(a,t)\;da$ is the total mass in pool *i*, and $x(t)={\left(x_{1}(t),\ldots ,x_{d}(t)\right)}^{⊤}$ solves (3.1).

The extended McKendrick–von Foerster equation is then given by

3.3 $$\frac{\partial p_{ik}}{\partial t}=\sum_{\;j=1}^{d}b_{ij}(t)p_{\;jk}\;\text{for}\;k\neq i$$

and

3.4 $$\frac{\partial p_{ii}}{\partial t}+\frac{\partial p_{ii}}{\partial a}=\sum_{\;j=1}^{d}b_{ij}(t)p_{\;ji}\;\text{for}\;k=i$$

with boundary condition

$$p_{ii}(0,t)=s_{i}(t)\;\text{and}\;p_{ik}(0,t)=0\;\text{for all}\;k\neq i.$$

Note that the first part (3.3) of this extended equation does not have a term of the form ∂*p*_*ik*_/∂*a*, since *p*_*ik*_(*a*, *t*) for i≠k is not ageing (it describes only the times spent in other pools) and thus it is only exposed to the homogeneous dynamics given by $\;\dot{\;x}=B(t)x$.

The mass densities *p*_*ik*_(*a*, *t*) relate to the above pool occupancy times *a*_*ik*_(*t*) of interest via

3.5 $$a_{ik}(t)=\frac{\int_{0}^{\infty }a\;p_{ik}(a,t)\;da}{\sum_{\ell =1}^{d}\int_{0}^{\infty }p_{i\ell }(a,t)\;da}.$$

Then the pool occupancy times *a*_*ik*_(*t*) satisfy the differential equation

$${\dot{a}}_{ii}=1+\frac{\sum_{\;j=1}^{d}(a_{\;ji}-a_{ii})b_{ij}(t)x_{j}(t)-a_{ii}s_{i}(t)}{x_{i}(t)}$$

and

$${\dot{a}}_{ik}=\frac{\sum_{\;j=1}^{d}(a_{\;jk}-a_{ik})b_{ij}(t)x_{j}(t)-a_{ik}s_{i}(t)}{x_{i}(t)}\;\text{for }k\neq i.$$

We call this differential equation the *pool mean age equation*, as a refinement of the mean age equation (3.2) introduced in [16]. We note that the pool mean age equation can be written more compactly as a matrix differential equation. More precisely, the matrix *A*(*t*) = (*a*_*ij*_(*t*))_*i*,*j*=1, … ,*k*_ satisfies the matrix differential equation

3.6 $$\dot{A}={\mathrm{Id}}_{d}+X{\left(t\right)}^{-1}(B(t)X(t)-\dot{X}(t))A$$

where $X(t)\;:=\mathrm{diag}(x_{1}(t),x_{2}(t),\;\ldots ,\;x_{d}(t))$ and $\dot{X}(t)\;:=\mathrm{diag}$ $\left({\dot{x}}_{1}(t),{\dot{x}}_{2}(t),\;\ldots ,\;{\dot{x}}_{d}(t))\mathrm{diag}({\dot{x}}_{1}(t),{\dot{x}}_{2}(t),\;\ldots ,\;{\dot{x}}_{d}(t)\right)$.

We refer to appendix B for a derivation of the pool mean age equation, and note that it is possible to compute higher-order moments of the pool occupancy times distributions, similarly to the derivations in [18] for the whole system.

We now combine the time-dependent matrix *A*(*t*) (which contains the pool occupancy times of particles spent in different pools) with the mass available in the compartmental system (described either as vector $x(t)={\left(x_{1}(t),\;\ldots ,\;x_{d}(t)\right)}^{⊤}$ or as matrix $X(t)=\mathrm{diag}(x_{1}(t),x_{2}(t),\;\ldots ,\;x_{d}(t))$. Doing so, we get the average time spent in pool *k* at time *t*, given by

3.7 $$M_{k}(t)\;:=\frac{\sum_{i=1}^{d}a_{ik}(t)x_{i}(t)}{\sum_{i=1}^{d}x_{i}(t)},$$

which can be rewritten as a *d*-dimensional (row) vector

3.8 $$M(t)\;:=\frac{\left(1,\;\ldots ,\;1)X(t)A(t\right)}{(1,\;\ldots ,\;1)X(t){\left(1,\;\ldots ,\;1\right)}^{⊤}}.$$

We consider now specifically the autonomous case, which is compartmental systems of the form

3.9 $$\;\dot{\;x}=Bx+s,$$

and if we are in equilibrium, then $\;\dot{\;x}=0$, and thus *x*(*t*) = *x* = −*B*^−1^*s*. We note that in this case, also $\;\dot{\;A}=0$, and (3.6) then implies that

3.10 $$A(t)=A=-X^{-1}B^{-1}X.$$

Using this, one gets

3.11 $$M(t)=M=\frac{-(1,\;\ldots ,\;1)B^{-1}X}{(1,\;\ldots ,\;1)X{\left(1,\;\ldots ,\;1\right)}^{⊤}}.$$

In particular, the mean age (i.e. the average time since entering the system) is then given by

3.12 $$\frac{-(1,\;\ldots ,\;1)B^{-1}X{\left(1,\;\ldots ,\;1\right)}^{⊤}}{(1,\;\ldots ,\;1)X{\left(1,\;\ldots ,\;1\right)}^{⊤}}=-(1,\;\ldots ,\;1)B^{-1}\gamma ,$$

where *γ* = (*γ*_1_, … , *γ*_*d*_)^*T*^, given by

$$\gamma_{i}=\frac{x_{i}}{\sum_{\;j=1}^{d}x_{j}}\;\text{for all }\;i\in \{1,\;\ldots ,\;d\},$$

describes how mass is distributed when the system is in equilibrium. Note that $\sum_{i=1}^{d}\gamma_{i}=1$. This confirms the formula established in [16], §2, formula (3).

## 4. Pool transit times

We now turn our attention to the transit times for the pools of our compartmental system. These times describe the average times spent in certain pools of particles leaving the system at a particular time. More precisely, the average amount of time a particle leaving the system at time *t* has spent in pool *k* is given by

4.1 $$R_{k}(t)=\frac{\sum_{i=1}^{d}a_{ik}(t)x_{i}(t)\sum_{\;j=1}^{d}b_{\;ji}(t)}{\sum_{i=1}^{d}x_{i}(t)\sum_{\;j=1}^{d}b_{\;ji}(t)},$$

and using the notation developed in the previous section, the *d*-dimensional (column) vector *R*(*t*) consisting of these times can be written as

4.2 $$R(t)=\frac{A{\left(t\right)}^{⊤}X{\left(t\right)}^{⊤}B{\left(t\right)}^{⊤}{\left(1,\;\ldots ,\;1\right)}^{⊤}}{(1,\;\ldots ,\;1)B(t)X(t){\left(1,\;\ldots ,\;1\right)}^{⊤}}.$$

As already hinted at in §2, this quantity is difficult to understand even for simple examples, and mainly accessible only via numerical computations. We note, however, that in the autonomous case, given by (3.9), all information is basically contained in the matrix inverse *B*^−1^.

To see this, we can use, as above, (3.10), and $x=X{\left(1,\;\ldots ,\;1\right)}^{⊤}=-B^{-1}s$ (see text below (3.9)), and get

4.3 $$\begin{array}{rl} R(t)=R & =\frac{-X{(B^{-1})}^{⊤}B^{⊤}{\left(1,\;\ldots ,\;1\right)}^{⊤}}{(1,\;\ldots ,\;1)s}\\ & =-\frac{B^{-1}s}{(1,\;\ldots ,\;1)s}=-B^{-1}\beta , \end{array}$$

where $\beta ={\left(\beta_{1},\;\ldots ,\;\beta_{d}\right)}^{⊤}$ describes the fractions of particles that enter the system from outside into the different pools, i.e.

4.4 $$\beta_{i}=\frac{s_{i}}{\sum_{i=1}^{d}s_{i}}.$$

Note that this corresponds to and extends the formula established in [16], §2, formula (2).

## 5. Pool occupancy times and pool transit times for the Carnegie–Ames–Stanford approach model

In this section, we apply the above theory of pool occupancy times and pool transit times to a system modelling terrestrial carbon under climate change. We consider a modification of the original Carnegie–Ames–Stanford approach (CASA) model that was first introduced in [24], given by a nine-dimensional linear differential equation

$$\;\dot{\;x}=B(t)x+s(t),$$

modelling nine different carbon pools: the first three pools represent plant biomass, the second three pools represent litter, and the last three pools correspond to soil organic matter. We note that this is the same model analysed in our earlier paper [16], where we studied the mean age of carbon and the transit time for the whole system. With the theory developed in this paper, we are able to see how these times are decomposed into the nine different pools.

The time-dependence of both *B*(*t*) and *s*(*t*) is due to an increase of atmospheric CO_2_ over time. This increase of CO_2_ leads to a rise of mean global temperatures which increases the carbon loss rates in the litter and soil pools, decreasing the corresponding components in the matrix *B*(*t*), and also has an effect on *s*(*t*): due to carbon dioxide fertilization, CO_2_ directly increases the carbon inputs *s*(*t*).

We note that the model does not depend on time until the year 1850, and the model strongly depends on time after the year 1850, for the above reasons, but the time-dependence weakens around the year 2150, and the model is asymptotically autonomous in the limit *t* → ∞.

The matrix *B*(*t*) is given by

$$B(t)=\left(\begin{array}{ccccccccc} b_{11} & 0 & 0 & 0 & 0 & 0 & 0 & 0 & 0\\ 0 & b_{22} & 0 & 0 & 0 & 0 & 0 & 0 & 0\\ 0 & 0 & b_{33} & 0 & 0 & 0 & 0 & 0 & 0\\ b_{41} & b_{42} & 0 & b_{44}\eta (t) & 0 & 0 & 0 & 0 & 0\\ b_{51} & b_{52} & 0 & 0 & b_{55}\eta (t) & 0 & 0 & 0 & 0\\ 0 & 0 & b_{63} & 0 & 0 & b_{66}\eta (t) & 0 & 0 & 0\\ 0 & 0 & 0 & b_{74}\eta (t) & b_{75}\eta (t) & b_{76}\eta (t) & b_{77}\eta (t) & b_{78}\eta (t) & b_{79}\eta (t)\\ 0 & 0 & 0 & 0 & b_{85}\eta (t) & b_{86}\eta (t) & b_{87}\eta (t) & b_{88}\eta (t) & 0\\ 0 & 0 & 0 & 0 & 0 & 0 & b_{97}\eta (t) & b_{98}\eta (t) & b_{99}\eta (t) \end{array}\right),$$

where the decay rates of the last six pools, i.e. the litter and soil pools, depend on time, and this is modelled by multiplication of a scalar function *η*(*t*). The coefficients *b*_*ij*_ are given as follows: *b*_11_ = −0.67, *b*_22_ = −0.2, *b*_33_ = −0.04, *b*_41_ = 0.5092, *b*_42_ = 0.0260, *b*_44_ = −2.5, *b*_51_ = 0.1608, *b*_52_ = 0.1740, *b*_55_ = −0.4, *b*_63_ = 0.04, *b*_66_ = −0.25, *b*_74_ = 1.1250, *b*_75_ = 0.1530, *b*_76_ = 0.06, *b*_77_ = −0.7, *b*_78_ = 0.0103, *b*_79_ = 0.0002, *b*_85_ = 0.042, *b*_86_ = 0.07, *b*_87_ = 0.3525, *b*_88_ = −0.023, *b*_97_ = 0.0045, *b*_98_ = 0.0001 and *b*_99_ = −0.0004. Note that the scalar function *η* is only multiplied to the rates in the last six pools, so the pools presenting plant biomass are assumed to show autonomous behaviour.

The input vector *s*(*t*) modelling the uptake of carbon by the terrestrial part of the carbon cycle is given by

$$s(t)=(s_{1}(t),s_{2}(t),s_{3}{\left(t),0,\;\ldots ,\;0\right)}^{⊤}.$$

**Figure 7.**
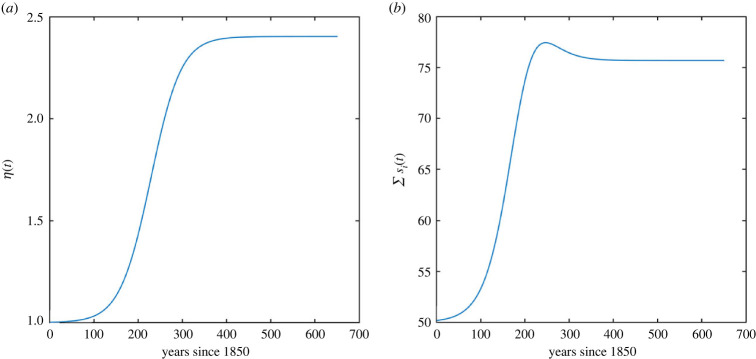
Time-dependence in the CASA model: (*a*) scalar factor *η*(*t*) that is multiplied to the coefficients in the litter and soil pools and (*b*) total carbon inputs $\sum_{i=1}^{3}s_{i}(t)$ per unit of time.

All the details of this model are presented in appendix C (see also [16]), and we refer to figure 7 for an illustration of the non-autonomous factors *η*(*t*) and $\sum_{i=1}^{3}s_{i}(t)$.

**Figure 8.**
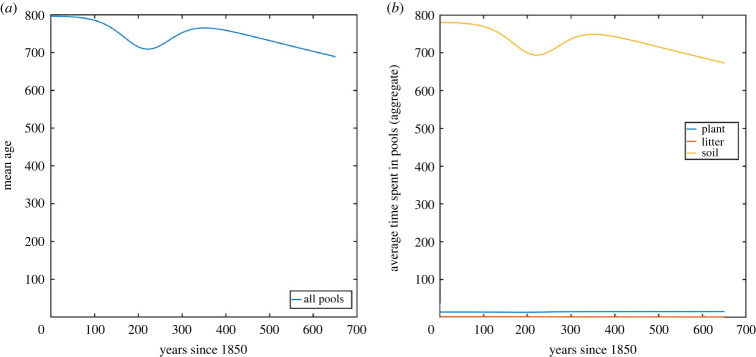
Average time spent by carbon in the CASA model: (*a*) in the whole system (mean age) and (*b*) in the three different types of pools (plant, litter and soil).

**Figure 9.**
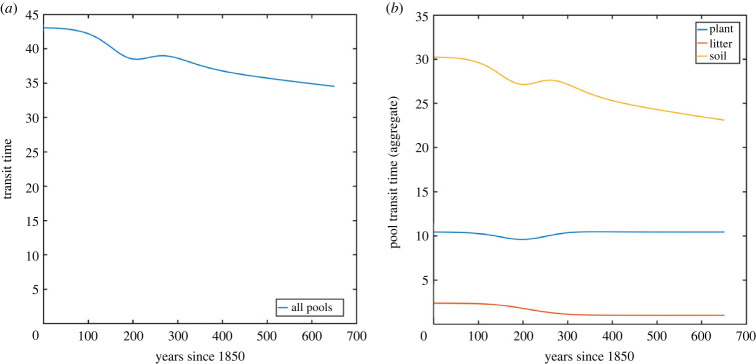
Transit times of carbon in the CASA model: (*a*) aggregated for the whole system and (*b*) aggregated for the three different types of pools (plant, litter and soil).

We have calculated numerically the pool occupancy times and transit times for all nine pools from the year 1850 until 2500, and we present the results in both figures and tables. In figures 8 and 9, the pool occupancy times and transit times have been aggregated for all pools (on the left), which corresponds to the analysis in our earlier paper [16]. On the right side in these figures, we present aggregations of the average time spent in different pools, and aggregations of the pool transit times, both with respect to the different types of pools (plant, litter and soil).

We do not present the pool occupancy times and transit times graphically for all nine pools. Instead, we demonstrate the numerical values in tables 1 and 2 for the years 1850, 1950, 2050, 2150, 2250, 2350 and 2450. Moreover, in table 3, we present the relative contributions of each pool to the transit time for the whole system.

**Table 1.** Average times spent in the nine pools of the CASA model, starting from the year 1850.

	plant pools			litter pools			soil pools		
year	pool 1	pool 2	pool 3	pool 4	pool 5	pool 6	pool 7	pool 8	pool 9
1850	0.33	1.44	12.25	0.07	0.70	1.19	1.02	30.83	748.20
1950	0.33	1.43	12.16	0.07	0.69	1.17	1.00	30.21	737.93
2050	0.32	1.40	11.89	0.06	0.58	0.94	0.85	24.78	673.77
2150	0.29	1.34	13.21	0.05	0.42	0.65	0.67	16.92	719.27
2250	0.29	1.32	13.55	0.04	0.38	0.58	0.61	14.23	727.48
2350	0.29	1.32	13.58	0.04	0.37	0.57	0.59	13.67	700.94
2450	0.28	1.32	13.63	0.04	0.36	0.55	0.57	13.34	672.73

**Table 2.** Transit times for the nine pools of the CASA model, starting from the year 1850.

	plant pools			litter pools			soil pools		
year	pool 1	pool 2	pool 3	pool 4	pool 5	pool 6	pool 7	pool 8	pool 9
1850	0.49	1.70	8.25	0.12	0.94	1.32	0.77	17.50	11.96
1950	0.50	1.70	8.07	0.12	0.92	1.28	0.75	17.07	11.77
2050	0.50	1.70	7.40	0.09	0.72	0.98	0.60	14.75	11.77
2150	0.49	1.70	8.17	0.05	0.44	0.63	0.39	10.31	16.43
2250	0.49	1.70	8.28	0.05	0.40	0.56	0.33	7.69	17.27
2350	0.49	1.70	8.25	0.05	0.39	0.55	0.33	7.41	16.54
2450	0.49	1.70	8.25	0.05	0.39	0.55	0.33	7.39	15.76

**Table 3.** Proportion of each of the nine pools to the transit time for the whole system.

	plant pools			litter pools			soil pools		
year	pool 1 (%)	pool 2 (%)	pool 3 (%)	pool 4 (%)	pool 5 (%)	pool 6 (%)	pool 7 (%)	pool 8 (%)	pool 9 (%)
1850	1.14	3.95	19.17	0.27	2.18	3.07	1.78	40.65	27.79
1950	1.18	4.02	19.13	0.28	2.18	3.03	1.78	40.49	27.92
2050	1.30	4.41	19.21	0.23	1.86	2.53	1.56	38.31	30.57
2150	1.27	4.40	21.16	0.14	1.14	1.63	1.01	26.69	42.55
2250	1.34	4.62	22.51	0.14	1.08	1.52	0.90	20.92	46.97
2350	1.38	4.76	23.10	0.14	1.10	1.55	0.92	20.75	46.30
2450	1.41	4.87	23.63	0.14	1.13	1.58	0.93	21.16	45.13

Firstly, the numerical data shows that carbon spends most of the time in the soil pools, and in particular in pools 8 and 9. This is not so surprising, since they have the slowest rates and for this reason, mass stays for a very long time in these pools. Table 3 shows that such a dominance can also be seen when looking at the transit times. However, while pool 8 is the dominating pool in 1850 (with respect to the transit time), this role is exchanged with pool 9. The reason for this is that the mass goes down slower in pool 9, and the average times in this pool take longer to adapt (which can also be seen from table 1, in particular in comparison with pool 8). Since transit times correspond to the age of carbon that leaves the system, this delay effect means that pool 9 still releases carbon with a higher age for a significant amount of time.

The numerical data presented in the figures demonstrate a non-monotonic behaviour of the average times spent by the carbon, and of transit times, which was already observed for the aggregated figures in our earlier paper [16]. The detailed analysis on the level of pools presented in this paper shows that this non-monotonicity is observed in the plant and the soil pools, while the litter pools show monotone behaviour. The non-monotonicity in the plant pools seems particularly surprising since the rates in the plant pools do not depend on time. As outlined earlier in §2.2, the reasons for non-monotonic behaviour can already be difficult to explain in a two-dimensional context, and we stress that such a behaviour is a combination of different phenomena, some of which have a decreasing effect on the transit time, while others work in the other direction. We first note that a decrease in average times in certain pools can come from two factors that are both present in the CASA model: growing inputs via *s*(*t*) and increasing decomposition rates given by *η*(*t*) (see also figure 7). Moreover, it is clear that a decrease in the average times should lead to a decrease in the transit times as a consequence. Overall, when looking at the dominating trend in the long-term behaviour, we observe a decrease of both average times and transit times, and this fits well with the intuitive explanation above. However, the above-mentioned non-monotonic behaviour seems to be more difficult to grasp. It may be tempting to attribute this to a temporary change in the monotonicity of the inputs *s*(*t*) (as displayed in figure 7*b*), but as we have seen in numerical simulations not presented here, this phenomenon can also be observed in the context of constant inputs. We believe that the fact that the carbon from the three plant pools leaves with different rates to the other pools has an increasing effect on the transit times (at certain times) in this particular example. To understand this better, table 1 reveals that the average time spent in pool 1 decreases over time while the average time spend in pool 3 increases (after they decreased for a while). This contrary behaviour happens since carbon from the first three pools is distributed with different rates to the other pools, and the behaviour in these other pools is quite contrasting due to the overall time-dependence (as outlined earlier when we explained that pool 9 becomes the dominating pool in its contribution to the overall transit time). For this reason, this phenomenon comes from the fact that the plant pools are higher dimensional, and in particular, this effect cannot happen in a situation where there is only one plant pool. This demonstrates that a lower-dimensional model (perhaps a three-dimensional model for plants, litter and soil) would not capture this particular non-monotone phenomenon. We note that it is difficult to make these arguments more precise and generalizable to other models. Overall, non-autonomous compartmental models can, in contrast to time-independent models, show a much more complicated behaviour that is often difficult to understand.

Finally, we point out that non-monotonicity can also be observed when looking at the mass storage in each of the pools and in the entire terrestrial system, see table 4. The mass in the entire system increases until about the year 2050 and then decreases well below the level of 1850. The reason for the initial increase in carbon storage is given by the fact that the inputs increase, modelled by *s*(*t*), but on a longer timescale, the decreasing effect given by the speed-up of the decomposition rates in the litter and soil pools, modelled by *η*(*t*), dominates. We note that the carbon storage in the first three pools (the plant pools) is not affected by the decline and increases over time due an increase in atmospheric CO_2_.

**Table 4.** Mass storage in each of the nine pools, and total storage.

	plant pools			litter pools			soil pools			
year	pool 1	pool 2	pool 3	pool 4	pool 5	pool 6	pool 7	pool 8	pool 9	all pools
1850	24.63	85.00	412.50	5.90	46.88	66.00	38.39	874.85	598.13	2152.27
1950	26.15	89.78	427.72	6.07	48.01	66.35	39.00	880.18	598.23	2181.47
2050	36.21	123.71	562.03	6.03	47.85	63.04	38.99	897.54	599.07	2374.46
2150	37.66	130.09	632.34	4.00	31.89	45.04	27.04	652.04	592.75	2152.86
2250	37.30	128.75	625.42	3.73	29.65	41.80	24.43	559.70	576.04	2026.82
2350	37.28	128.67	624.47	3.72	29.54	41.59	24.25	552.49	559.06	2001.07
2450	37.28	128.67	624.41	3.72	29.53	41.58	24.24	552.02	543.43	1984.88

## 6. Discussion

Both to understand the implications of observations of the dynamics through time of carbon storage in different compartments [25,26] and to understand dynamics of future carbon storage, the effect of changes through time in fluxes between pools on carbon storage is needed. The understanding for systems with parameters that are constant in time is well developed, and recently, there have been results regarding transit times for systems where fluxes (parameters) change through time [16–18]. We have built on these recent contributions and have developed refinements in this paper that provide understanding of the transit time behaviour on the pool level.

As has been emphasized [27] a truly important question is to be able to predict future trends in carbon storage based on combining current estimates of how fluxes are changing through time and observations of the sizes of current pools. The simplest prediction of future changes in storage would be to extrapolate from current trends. Our analysis of models incorporating time-dependence shows how complicated even predictions based on simple models can be. The key overall qualitative result emerging from our analysis is that even when the flow rates between pools change monotonically through time, that the average times of the carbon stored in different pools and the pool transit times do not change monotonically. We have illustrated this with a specific example in §5, but our relatively general model here is not an attempt to make specific predictions, as we have not incorporated all the details in current observations [27]. However, the heuristic conclusions we present here are key when interpreting data and choosing management options.

We will emphasize the importance of this result by two other qualitative interpretations. In addition to the view based on prediction, there are two ways to think about the implication of this result for drawing conclusions from observations of changes in mean ages (which are related to overall storage) of carbon in different pools. First, non-monotonic changes in the ages of carbon, or transit times, in different pools do not imply that fluxes are changing non-monotonically. As a final point, in the absence of a careful model, it would be, in the face of changing fluxes, in general incorrect to extrapolate even the direction of future changes in transit times or mean ages in different pools based on current changes.

Given the surprising nature of our general predictions, it is important to provide some intuition why this non-monotonicity can emerge. From a heuristic standpoint, these potential difficulties in understanding changes though time for a system with changing parameters are related to the idea that non-autonomous systems make explicit the difference between future changes and changes that have led to the current state of a dynamical system: the pullback attractor and the forward attractor, which are the precise mathematical terms for these two different aspects, are different objects [10].

An obvious next step is to take our general results and apply them to understand more detailed specific information about fluxes at either a global or more restricted scale. Only through this kind of analysis can we demonstrate how important the issues we raise here are from a quantitative standpoint. In this vein, the results here are important for understanding the efficacy of any actions taken to increase carbon storage. If future trends are not predicted by current trends when fluxes are changing through time, careful models combined with careful observations rather than focusing on observed trends is the only appropriate way to judge.

## Data Availability

The Matlab codes are provided in electronic supplementary material [28].

## Appendix A. Non-positivity of the matrix *B*^−1^

We demonstrate now that the inverse *B*^−1^ of a matrix *B* modelling a compartmental system is non-positive. Consider first the matrix $D=-B^{⊤}$. Due to the properties of the matrix *B* (mentioned in §2.1), we see that *d*_*ii*_ > 0, and that for *i* ≠ *j* the entries *d*_*ij*_ ≤ 0. Moreover, $\sum_{j}d_{ij}\geq 0$. The following demonstration is based on ([29], section 2.5). Firstly, note that *D* is a Z-matrix, which is defined to be a matrix with non-positive off-diagonal entries. We now show that *D* is also an M-matrix, which is a Z-matrix with eigenvalues whose real parts are non-negative. This would imply that *B*^−1^ is non-negative, since inverse of an M-matrix is non-negative. One condition that would imply that the Z-matrix *D* is an M-matrix would be if there is a positive vector *x* such that *Dx* is also a positive vector. By the sign pattern of the entries in *D* and that the row sums are non-negative with at least one row sum positive, we choose the vector *x* to be the vector with *x*_*i*_ = 1 for those rows *i* where the row sum is positive and $x_{i}=1+\varepsilon$ for those rows *i* where the row sum is zero. It is clear that for sufficiently small ε>0, we get a vector *x* where *Dx* > 0 showing that *D*^−1^ ≥ 0. This is equivalent to −*B*^−1^ ≥ 0.

## Appendix B. Derivation of the pool mean age equation

We present two different approaches to derive the pool mean age equation that we have introduced in §3. As explained in this section, one can use the extended McKendrick–von Foerster equation, and using $\sum_{k=1}^{d}\int_{0}^{\infty }a(\partial p_{ik}/\partial a)(a,t)\;da=-x_{i}(t)$ (which follows from integration by parts), we first get (for *k* = *i*)

$$\begin{array}{rl} {\dot{a}}_{ii}(t) & =\frac{x_{i}(t)\int_{0}^{\infty }a(\partial p_{ii}/\partial t)(a,t)\;da-a_{ii}(t)x_{i}(t)\;{\dot{\;x}}_{i}(t)}{x_{i}^{2}(t)}\\ & =\frac{x_{i}(t)\int_{0}^{\infty }a\left(-(\partial p_{ii}/\partial a)(a,t)+\sum_{\;j=1}^{d}b_{ij}(t)p_{\;ji}(a,t)\right)\;da-a_{ii}(t)x_{i}(t)\;{\dot{\;x}}_{i}(t)}{x_{i}^{2}(t)}\\ & =\frac{x_{i}^{2}(t)+\sum_{\;j=1}^{d}b_{ij}(t)x_{i}(t)\int_{0}^{\infty }ap_{\;ji}(a,t)\;da-a_{ii}(t)x_{i}(t)\;{\dot{\;x}}_{i}(t)}{x_{i}^{2}(t)}\\ & =1+\frac{\sum_{\;j=1}^{d}b_{ij}(t)x_{j}(t)a_{\;ji}(t)-a_{ii}(t)\left(\sum_{\;j=1}^{d}b_{ij}(t)x_{j}(t)+s_{i}(t)\right)}{x_{i}(t)}\\ & =1+\frac{\sum_{\;j=1}^{d}(a_{\;ji}(t)-a_{ii}(t))b_{ij}(t)x_{j}(t)-a_{ii}(t)s_{i}(t)}{x_{i}(t)}. \end{array}$$

In case of k≠i, we arrive at

$$\begin{array}{rl} {\dot{a}}_{i}^{k}(t) & =\frac{x_{i}(t)\int_{0}^{\infty }a(\partial p_{ik}/\partial t)(a,t)\;da-a_{ik}(t)x_{i}(t)\;{\dot{\;x}}_{i}(t)}{x_{i}^{2}(t)}\\ & =\frac{x_{i}(t)\int_{0}^{\infty }a\sum_{\;j=1}^{d}b_{ij}(t)p_{\;jk}(a,t)\;da-a_{ik}(t)x_{i}(t)\;{\dot{\;x}}_{i}(t)}{x_{i}^{2}(t)}\\ & =\frac{\sum_{\;j=1}^{d}b_{ij}(t)x_{i}(t)\int_{0}^{\infty }ap_{\;jk}(a,t)\;da-a_{ik}(t)x_{i}(t)\;{\dot{\;x}}_{i}(t)}{x_{i}^{2}(t)}\\ & =\frac{\sum_{\;j=1}^{d}b_{ij}(t)x_{j}(t)a_{j}^{k}(t)-a_{ik}(t)\left(\sum_{\;j=1}^{d}b_{ij}(t)x_{j}(t)+s_{i}(t)\right)}{x_{i}(t)}\\ & =\frac{\sum_{\;j=1}^{d}(a_{j}^{k}(t)-a_{ik}(t))b_{ij}(t)x_{j}(t)-a_{ik}(t)s_{i}(t)}{x_{i}(t)}. \end{array}$$

Alternatively, one can derive the pool mean age equation using a discretization argument. For k≠i, we get

$$\begin{array}{r} a_{ik}(t+\Delta t)\approx \frac{a_{ik}(t)(x_{i}(t)+b_{ii}(t)x_{i}(t)\Delta t)+\sum_{\;j\neq i}a_{j}^{k}(t)b_{ij}(t)x_{j}(t)\Delta t}{x_{i}(t+\Delta t)}. \end{array}$$

Hence,

$$\frac{a_{ik}(t+\Delta t)-a_{ik}(t)}{\Delta t}\approx \frac{a_{ik}(t)x_{i}(t)}{x_{i}(t+\Delta t)\Delta t}-\frac{a_{ik}(t)}{\Delta t}+\underset{\to (\sum_{\;j=1}^{d}a_{\;jk}(t)b_{ij}(t)x_{j}(t)/x_{i}(t))\mathrm{as}\Delta t\to 0}{\underbrace{\frac{\sum_{\;j=1}^{d}a_{\;jk}(t)b_{ij}(t)x_{j}(t)\Delta t}{x_{i}(t+\Delta t)\Delta t}}},$$

so the third term is understood. We now look at the first two terms and get

$$\begin{array}{rl} & \frac{a_{ik}(t)x_{i}(t)}{x_{i}(t+\Delta t)\Delta t}-\frac{a_{ik}(t)}{\Delta t}=\frac{a_{ik}(t)}{x_{i}(t+\Delta t)\Delta t}(x_{i}(t)-x_{i}(t+\Delta t))\\ & =-\frac{a_{ik}(t)}{x_{i}(t+\Delta t)}\frac{x_{i}(t+\Delta t)-x_{i}(t)}{\Delta t}\\ & \to -\frac{a_{ik}(t)}{x_{i}(t)}\left(\sum_{\;j=1}^{d}b_{ij}(t)x_{j}(t)+s_{i}(t)\right)\;\text{as }\Delta t\to 0. \end{array}$$

The case *i* = *k* can be treated similarly.

## Appendix C. Additional details on the modified CASA model

We present additional details on the CASA model. The CASA model crucially depends on the atmospheric carbon dioxide concentration in parts per million, given by

$$x_{a}(t)=1715\exp\left(\frac{0.0305t}{\left(1715+\exp(0.0305t)-1\right)}\right),$$

where *t* is years since the year 1850. This represents a plausible time course of atmospheric CO_2_ from year 1850 (*t* = 0) to 2500 (*t* = 650) under a zero-mitigation, business as usual global change scenario [30].

The effect of CO_2_ on mean global temperatures is modelled as

$$T_{s}(t)=T_{s0}+\frac{\sigma }{\ln(2)}\ln\left(\frac{x_{a}(t)}{285}\right),$$

where *T*_*s*0_ = 15 is the mean land surface temperature in 1850, and *σ* is the sensitivity of global temperatures to *x*_*a*_(*t*). We chose an upper extreme of *σ* = 4.5 based on the literature because the resulting simulation emphasizes well the interplay between increased carbon input rates and carbon loss rates [31]. Changes in carbon input rates are simulated using

$$s(t)=(s_{1}(t),s_{2}(t),s_{3}(t),0,0,0,0,0,0),$$

with *s*_*i*_(*t*) = *f*_*i*_*αs*_0_(1 + *β*(*x*_*a*_(*t*), *T*_*s*_(*t*))ln(*x*_*a*_(*t*)/285)), where *s*_0_ = 100, *f*_*i*_ = 0.33 is the proportion of carbon input going to the different carbon pools, *α* = 0.5 is the proportion of gross primary production that remains after respiration and *β* is the sensitivity of *s*(*t*) to *x*_*a*_(*t*) and *T*_*s*_(*t*), given by

$$\beta (x_{a}(t),T_{s}(t))=\frac{3\rho x_{a}(t)\Gamma (T_{s}(t))}{\left(\rho x_{a}(t)-\Gamma (T_{s}(t)))(\rho x_{a}(t)+2\Gamma (T_{s}(t))\right)},$$

where *x* = 0.65 is the ratio of the intracellular CO_2_ to *x*_*a*_(*t*), and $\Gamma (T_{s}(t))$ is given by

$$\Gamma (T_{s}(t))=42.7+1.68(T_{s}(t)-25)+0.012{\left(T_{s}(t)-25\right)}^{2},$$

see [32]. The non-autononomous factor *η*(*t*) describing the changes in time of the loss rates for pools *i* = {3, … , 9} is given by

C 1 $$\eta (t)=\xi_{b}^{0.1T_{s}(t)-1.5},$$

where *ξ*_*b*_ = 2.

To define the model initial conditions we assume that *x*_*a*_(*t*) = *x*_*a*_(0) for all *t* < 0 and that *x*(0) has reached the positive equilibrium solution of the resulting system of autonomous equations. The model is then simulated forward from this initial condition using (C 1) as the forcing function. Under this simulated scenario, total land carbon increases then decreases over time. This would represent an initial net uptake of carbon from the atmosphere due to carbon dioxide fertilization followed ultimately by a net carbon loss from the land back to the atmosphere due to global warming.
